# Assessing the protective effect of rosiglitazone against electronic cigarette/tobacco smoke-induced blood–brain barrier impairment

**DOI:** 10.1186/s12868-019-0497-5

**Published:** 2019-04-04

**Authors:** Farzane Sivandzade, Luca Cucullo

**Affiliations:** 1 Department of Pharmaceutical Sciences, Texas Tech University Health Sciences Center, 1300 S. Coulter Street, Amarillo, TX 79106 USA; 2 Center for Blood-Brain Barrier Research, Texas Tech University Health Sciences Center, Amarillo, TX 79106 USA

**Keywords:** Rosiglitazone, Oxidative stress, Blood–brain barrier, Alternative, Tight junctions, Nrf2, PPRγ

## Abstract

**Background:**

Smoking (TS) and recently e-cigarettes (EC) vaping, have been associated with vascular endothelial dysfunction primarily relevant to oxidative stress, exposure to nicotine, and smoking-induced inflammation. It is accepted that both EC and TS enhance glucose intolerance and the risk of developing type-2 diabetes mellitus which is also one of the causes of blood–brain barrier (BBB) damage and the higher risk of cerebrovascular diseases. Recent studies have shown how Metformin, the first common antidiabetic drug, can protect the BBB integrity through enhancement of nuclear factor erythroid 2-related factor (Nrf2) activity. Herein, we investigated the role of rosiglitazone (RSG; family of thiazolidinedione class used oral anti-diabetic drug) in TS/EC-induced BBB impairment.

**Results:**

Although the exact mechanism of RSG is not fully understood, previous studies have revealed that RSG can promote counteractive protective mechanisms primarily associated with the enhancement of Nrf2 activity through activation of the peroxisome proliferator-activated receptor gamma. In line with these findings, our results show an increased expression of PPARy by RSG, enhancement of Nrf2 activity and BBB protection against TS/EC exposure including reduced inflammation, oxidative stress, tight junction downregulation and loss of BBB integrity.

**Conclusions:**

RSG could be considered as a promising therapeutic potential to prevent TS/EC induced cerebrovascular dysfunction and possibly other xenobiotic substances which may impact the BBB via oxidative stress-mediated effects. However, additional in vivo studies and clinical setting will be needed to validate our results and assess the full extent of RSG protective effects.

## Background

Tobacco smoke (TS) is a diverse mixture of over 4700 toxic, carcinogenic and mutagenic chemicals, as well as stable and unstable free radicals and reactive oxygen species (ROS) constituents. Nowadays, tobacco smoking (TS) usage causes approximately 6 million deaths per year throughout the world, so that more than 480,000 deaths each year happens in the United States (US) and about 41,000 deaths out of 480,000 deaths result from second-hand smoke exposure. It must get into consideration that for every individual who dies due to smoking, at least 30 people suffer from a severe smoking-related illness [1–3]. Electronic cigarettes (EC) are battery-powered devices which vaporize through heat an e-liquid containing nicotine and a humectant (such as propylene glycol) which are then inhaled as a vapor. The heating process can lead to formation of reactive chemical species including ROS. Although EC may be considered as a safer product compared to TS (since they contain no tobacco and do not involve combustion, users may avoid several harmful constituents usually found in tobacco smoke, such as ash, tar, and carbon monoxide.), recent published data from side by side experiments investigating the impact of EC and TS clearly showed similar pro-oxidative effect [4, 5] and similar tendency to increase the risk of unwanted blood coagulation (through reduction of thrombin expression) [4]. Although Smokers are more likely to suffer from major illnesses such as heart disease (2–4 times higher), lung cancer (25 times higher), and type-2 diabetes mellitus [2, 3, 6], additional disorders associated with OS and inflammations have been associated with chronic smoking including rheumatoid arthritis, blindness, asthma, pneumonia, reduced fertility, hardening of the arteries, and enhanced risk of infections [2, 3, 7]. The content of TS and EC is associated with vascular endothelial dysfunction in a dose-dependent and causative manner [8, 9] which is primarily relevant to the content of reactive oxygen species (ROS), nicotine [3, 9], oxidative stress (OS) [10–13], blood–brain barrier (BBB) impairment [14, 15] and driven inflammation [16] leading to various cerebrovascular and neurological disorders including stroke [5, 17], amyotrophic lateral sclerosis (ALS), Alzheimer’s disease (AD), Parkinson’s disease (PD), and Huntington’s disease (HD) [18]. During pregnancy, TS can also impair the cerebrovascular development of the fetus [3]. In spite of a lot of debates over the benefits of prophylactic antioxidant treatments which may play a positive role in the reduction of adverse effects of smoke, it is required to detect biologic mechanisms by which TS exposure compromises health, identify biomarkers of injury and find more effective therapeutic agents to counteract the damaging effects and increased risks of vascular disorders associated with chronic smoking which persist for several years upon cessation [19, 20]. In the recent work of our group an additive release pattern of angiogenic and inflammatory factors by BBB endothelial cells in response to hyperglycemia (HG) with concomitant exposure to cigarette smoke extracts, suggesting the involvement of common pathogenic modulators of BBB impairment was confirmed [21]. Rosiglitazone (RSG), the thiazolidinedione compound that is well known to improve insulin resistance through regulating adiponectin gene expression, and used for the treatment of type-2 diabetes mellitus [22], is considered as a transcription factor peroxisome proliferator-activated receptor (PPARγ) agonist [23, 24]. Since chronic smoking and diabetes carry similar risks for cerebrovascular diseases and stroke, it is plausible that RSG can prevent/reduce BBB impairment promoted by the chronic TS/EC exposure. Although the exact mechanism of rosiglitazone is not fully understood, numerous studies have confirmed that RSG, may have the potential to ameliorate the oxidative damage [25]. Thus, the main aim of this study is investigating rosiglitazone’s (which is of the family of thiazolidinedione class used oral anti-diabetic drug) role in activation of counteractive antioxidative mechanisms to reduce TS and EC toxicity at the BBB. We found that RSG not only have protective effects through the activation of Nrf2, but also the antioxidant effect of RSG leads to the reduction of TS/EC-induced oxidative stress and loss of BBB integrity which might be through PPARγ-dependent or PPARγ-independent pathways.

## Methods

### Materials and reagents

Reagents and chemicals were purchased from Sigma-Aldrich (St. Louis, MO, USA) or Bio-rad laboratories (Hercules, CA, USA) and sterile culture wares were purchased from Fisher Scientific (Pittsburgh, PA, USA), Mouse primary brain microvascular endothelial cells (C57BL/6-mBMEC, #C57- 6023) and completed mouse endothelial cell medium kit (M1168) were obtained from Cell Biologics (Chicago, Illinois, USA). Methanol-free formaldehyde (Polysciences Inc. # 18814). Gel electrophoresis was carried out by using Mini-Protean^®^TGXTM gels 4–15% (#456–1084) from Bio-rad laboratories (Hercules, CA, USA). Fluorescein isothiocyanate (FITC)-dextran (3000–5000 MW; #FD4) and Rhodamine B isothiocyanate (RITC)—dextran (70,000 MW; #R9379) were obtained from Sigma-Aldrich (St. Louis, MO, USA). Rosiglitazone (RSG # A00183, MW: 357.4) was obtained from the Adipogen. The antibodies used in this study were purchased from the various sources: rabbit anti-PPARγ (#331500) from Invitrogen; rabbit anti-ZO-1 (#402200) from Life Technologies; rabbit anti-Nrf2 (#sc-722), mouse anti-NQO-1 (#sc-376023) and mouse anti-PECAM-1 (#sc-376764) from Santa Cruz Biotechnology. Sheep anti-mouse (#NA931) and Donkey anti-rabbit (#NA934) secondary antibodies were obtained from GE Healthcare (Piscataway, NJ, USA); goat anti-rabbit (#A11008, A21428) conjugated to Alexa Fluor^®^ 488 and 555 respectively and anti-mouse (#A11001, A21422) conjugated to Alexa Fluor^®^ 488 and 555 respectively from Invitrogen (Camarillo, CA, USA).

### Cell culture

Mouse primary brain microvascular endothelial cells (mBMEC-P3- C57BL/6J) were seeded on gelatin-coated cell culture flasks and glass chamber slides, cultured in supplemented medium (M1168) along with 10% FBS and maintained at 37 °C with 5% CO_2_ exposure [3]. The culture medium was changed every other day until the cells reached confluency. The monolayer integrity of mBMEC cells at confluency was confirmed by phase contrast microscopy and the expression of characteristic phenotypic markers.

### Soluble cigarette smoke extract preparation

Soluble TS and EC extract were prepared according to the FTC standard smoking protocol from 3R4F research cigarettes (35 mL puff volume, 2 s puff duration, 58 s intervals, 8 puffs per cigarette directly into phosphate buffered saline (PBS), equivalent to full flavor brands containing 9.4 mg tar and 0.726 mg nicotine per cigarette; University of Kentucky) and EC containing 2.4% nicotine [35 mL puff volume, 4 s puff duration, 17 s intervals, 8 puffs per cigarette directly into phosphate buffered saline (PBS)] using a Single Cigarette Smoking Machine (SCSM, CH Technologies Inc., Westwood, NJ, USA) following previously published methods [3, 9, 26]. TS/EC extract solutions were prepared fresh for each cycle and were then diluted to 5% concentrations in low serum media.

### Transwell cell culture set-up

mBMEC cells were seeded on the luminal side of Treated polyester transwell inserts (Corning^®^ Transwell^®^ polyester membrane cell culture inserts, 12 mm, 0.4 µm pore membrane insert; #3460) and grown in the supplemented culture medium, as mentioned above [3, 27]. The confluence and integrity of the cell monolayers were confirmed by phase contrast microscopy and trans-endothelial electrical resistance (TEER) measurement (baseline value ≈ 10 Ω cm^2^).

### Cell viability

MTT cell viability study was conducted in order to evaluate the appropriate RSG pretreatment dose for in vitro studies [3]. Briefly, cells cultured in a 96-wells plate were pre-treated with varying concentrations of RSG (20 µM to 2000 µM) for 24 h. Parallel RSG untreated controls were also evaluated for cell viability. Cells were stained with 10 µl of 5 mg/ml tetrazolium MTT (3-(4, 5-dimethylthiazolyl-2)-2, 5-diphenyltetrazolium bromide) for 3 h at 37 °C, after which 100 µl of DMSO (solubilizing agent) was added. The absorbance was read on a Bio-rad plate reader at a wavelength of 570 nm.

### Treatment (in vitro)

mBMEC cells were incubated in the low serum media containing supplemented culture medium (as mentioned earlier) and 1% FBS with no growth factors for 24 h. In all analyses, RSG was first dissolved in DMSO at a concentration of 100 mM, and then freshly diluted with supplemented culture medium to the appropriate concentrations [24]. The final concentration of DMSO was < 0.1%. Culture medium with DMSO served as the control in each RSG-treating experiment. The cells were then pretreated with RSG (20 µM based on our cell viability results) and also exposed to 5% TS/EC extract solution for 24 h [21]. Parallel controls (without EC/TS and/or RSG) were also provided.

### Preparation of protein extracts

In order to lyse cells to harvest the proteins either RIPA lysis buffer or subcellular protein fractionation kit for cultured cells (Thermo scientific, #78840) as per manufacturer’s guidelines was used, so that total proteins were collected by centrifugation at 14000 g for 30 min. Samples were then aliquoted and stored at − 80 °C until needed for protein expression analysis by western blotting.

### Western blotting

Proteins quantification was conducted using Pierce BCA Protein Assay Kit (Thermo Scientific, # 23225). Samples (15–30 μg for cell lysates) were then prepared according to the following method described in our previous lab report so that denatured samples were run on SDS-PAGE (4–15% gradient gel) and transferred to PVDF membranes for further blotting [3, 21]. The membranes were then washed with Tris-buffered saline (TBS) (10 mmol/l Tris–HCl, pH 7.4, 150 mmol/l NaCl) containing 0.1% Tween-20 (TBS-Tween), blocked for 1 h with TBS-Tween containing 5% non-fat dry milk, and incubated with primary antibodies prepared in TBS-Tween containing 5% bovine serum albumin (BSA) for overnight at 4 °C. The following day, for immunodetection, cells were washed and then incubated with the secondary antibody prepared in TBS-Tween containing 5% BSA for 2 h. Densities of the proteins’ s bands were analyzed using Image Studio Lite (LI-COR Biosciences—Ver 3.1) and expressed as either fold or percentage change over corresponding controls as previously reported by us [3]. β-actin was used to properly quantify protein expression levels independently from the treatment conditions.

### Immunofluorescence

mBMEC cells were seeded and grown in four-well chamber slides as mentioned previously. Cells were fixed in 16% methanol-free formaldehyde diluted 1 in 4 in 1X PBS, then washed and permeabilized using 0.02% Triton 100X. As previously described by us, [3] cells were then blocked with blocking buffer (5% goat serum in PBS) at 25 °C for about 1 h and incubated with primary antibodies prepared in blocking buffer for overnight at 4 °C. Cell were then washed and stained with Alexa Fluor^®^ 488 or 555 conjugated goat anti-rabbit or anti-mouse antibodies or vice versa at 25 °C and then mounted with DAPI [3]. Mounted slides then were examined under a digital epifluorescence and transmitted light microscope (EVOS™ FL Imaging System; Thermofisher). As negative controls we used cell slides stained with only secondary antibodies [3, 21].

### BBB integrity

BBB integrity was evaluated as previously described by us elsewhere [28]. Briefly, a mixture of labeled dextrans in PBS (FITC ~ 4 kDa, 10 mg/ml and RITC ~ 70 kDa, 10 mg/ml) were added to the luminal compartment of the transwells after 24 h of the last time of the treatment and exposure to TS/EC extract solution. Then, 50 µl of media sample was collected from the abluminal compartment at the time 0, 15 and 30 min and replaced with the equal volumes of fresh media in order to maintain appropriate sink conditions. Fluorescence was measured at specified excitation and emission wavelengths to determine the concentration of each fluorescent dye in the sample (FITC: 485 nm excitation/535 nm emission; RITC: 544 nm excitation/576 nm emission). Baseline permeability coefficients for FITC and RITC under normal conditions were approximately 2.1 × 10^−4^ cm/s and 5.8 × 10^−4^ cm/s. The permeability measurements were reported as the percentage of controls by considering the appropriate controls such as media samples without dextran and that from abluminal compartments of cell-free inserts with dextran added to the luminal compartment. In order to confirm the findings, we also measured TEER (Ω cm^2^) using EVOM 2 (World Precision Instruments, Sarasota, FL, USA). TEER measurements (baseline value) of cell-free inserts were used to determine the baseline values to subtract from the experimental readings.

### Measurement of intracellular ROS

To measure the amount of intracellular ROS, we used 2,7-dichlorodihydrofluorescein diacetate (2,7-DCFH-DA) as described elsewhere [24, 29]. Briefly, seeded mBMEC cells on the 96-well plate were washed with 100 μL/well of PBS once. 12.5 ul of 20,70-dichlorofluorescein diacetate (DCFHDA) 20 mM (in DMSO) was diluted in PBS to final concentration (25 µM). 100 µl of DCFHDA was added and incubated in the dark at 37 °C for 45 min. Cells were then washed thrice with PBS and 100 µl of PBS was added to each well. The intensity of dichlorofluorescein (DCF) fluorescent measurements in the supernatant were taken at specified excitation and emission wavelengths (485 nm excitation and 535 nm emission). The ROS generation was calculated as the percentage change over control.

### Statistical analysis

Data from all experiments were expressed as standard deviation (SD) and analyzed by one-way ANOVA using GraphPad Prism 6 Software Inc. (La Jolla, CA, USA). Post hoc multiple comparison tests were performed as suggested by the software. P values < 0.05 were considered statistically significant.

## Results

### Appropriate RSG dosing based on cell viability evaluated by MTT cytotoxicity

**Fig. 1:**
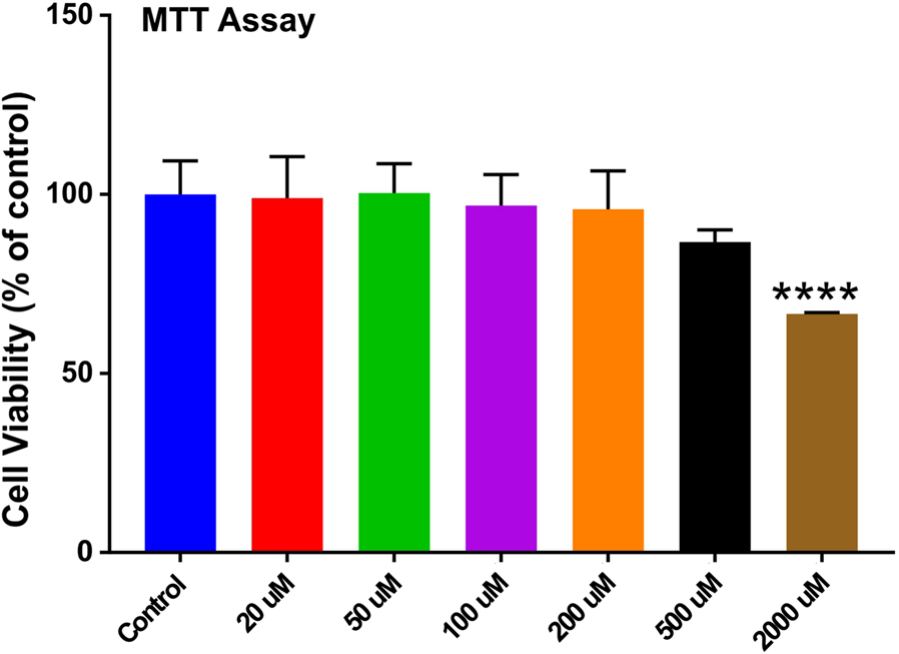
MTT cytotoxicity assay for RSG dose evaluation. n = 4 biological replicates; ****p < 0.0001 versus control

To evaluate appropriate RSG dosing based on its impact on cell toxicity and viability, MTT cytotoxicity assay was used. As shown in Fig. 1, compared to control, the viability of cells treated with different RSG doses (20 μM to 2000 μM) did not show any significant difference except for the highest concentration tested which resulted toxic to cell viability. Therefore, based on these results and literature evidence the RSG working dose for all our in vitro studies was finalized at 20 μM [23, 24].

### RSG significantly decreases intracellular ROS generated in response to TS/EC treatment as well as their pro-inflammatory activity

**Fig. 2:**
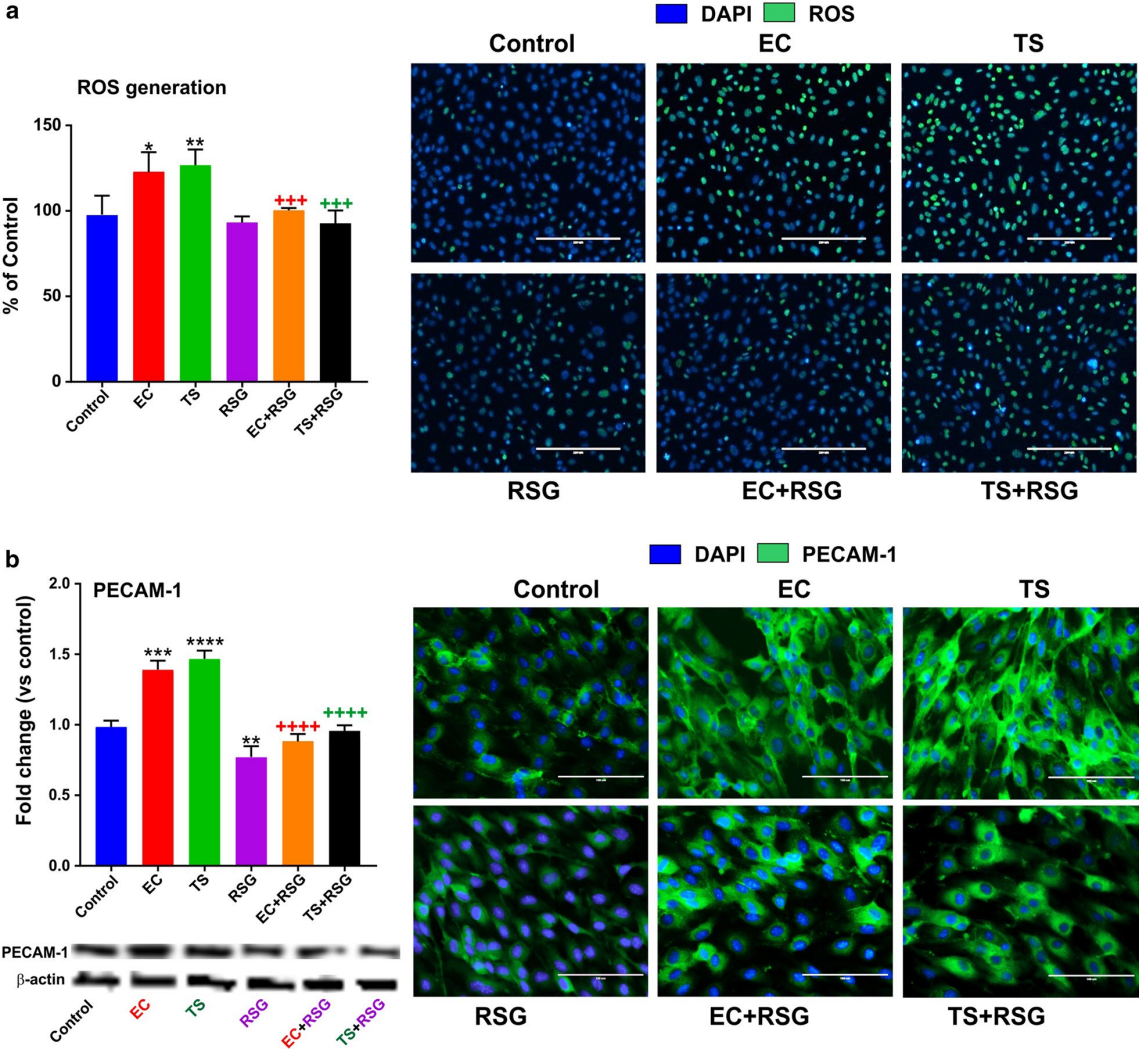
RSG decreases intracellular ROS generation and inflammation by TC/EC. **a** Intracellular ROS generation measured by flow cytometry. **b** Expression level of the inflammatory marker PECAM-1 which was increased in cells treated with TS/EC was downregulated by RSG. n = 3 to 4 biological replicates; *p < 0.05, **p < 0.01, ***p < 0.001, ****p < 0.0001, versus control; +++p < 0.001, ++++p < 0.0001, TS/EC versus TS/EC + RSG

**Fig. 3:**
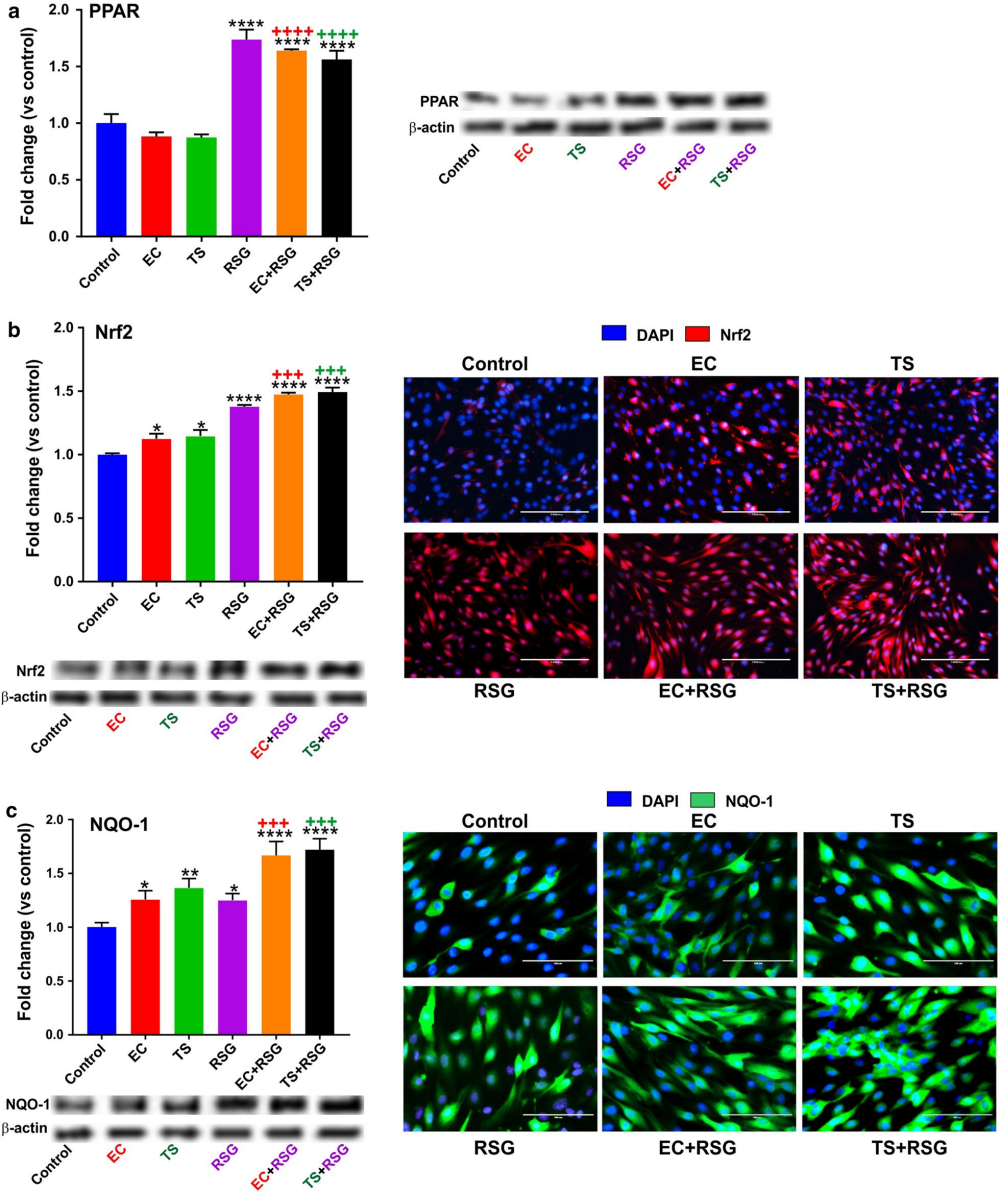
Protective effects of RSG against TS/EC-induced oxidative stress. **a** Immunofluorescence and Western blotting analysis emphasizing activation of the Nrf2 pathway. **b** Immunofluorescence and Western blotting analysis emphasizing the effect of RSG on activation of the transcription factor peroxisome proliferator-activated receptor (PPARγ). **c** Concurrently, the overexpression level of downstream detoxifying molecule NQO-1 in the cells including RSG was detected. n = 3 to 4 biological replicates. *p < 0.05, **p < 0.01, ****p < 0.0001, versus control; +++p < 0.001, ++++p < 0.0001, TS/EC versus TS/EC + RSG. WB analyses report protein/β-actin ratios

To measure whether the cell-damaging effect of TS/EC was related to oxidative stress, the intracellular ROS generation in both treated and untreated cells with RSG for 24 h was conducted. As shown in Fig. 2a, while a significant increase in intracellular ROS in the untreated TS/EC-exposed samples was observed. In addition to reduces oxidative stress, RSG also decreased TC/EC pro-inflammatory activity as demonstrated by the reduced expression of the inflammatory adhesion molecule PECAM-1 (see Fig. 2b). RSG treatment significantly decreased ROS level and the effect seems to parallel PPARγ upregulation and Nrf2 activation (see Fig. 3).

### RSG upregulates PPAR expression as well as NRF2 and its downstream effector NQO-1

The effect of TS/EC on the expression of the Nrf2 as a key antioxidant transcription factor, and also PPARγ was also evaluated. As shown in Fig. 3a the overall total expression levels of Nrf2 in TS/EC exposed cultures was slightly more than control. Figure 3 showed that treatment of the cells with RSG not only significantly stimulated the expression of PPARγ (Fig. 3a) but also increased the expression level of Nrf2 (Fig. 3b) as demonstrated by western blot and immunofluorescence analyses. Increased expression/activity of Nrf2 is also supported by measurements (both western blot and immunofluorescence analyses) of its downstream effector NQO-1 as demonstrated in Fig. 3c.

### RSG decreases TS/EC-induced endothelial inflammation and loss of barrier integrity

**Fig. 4:**
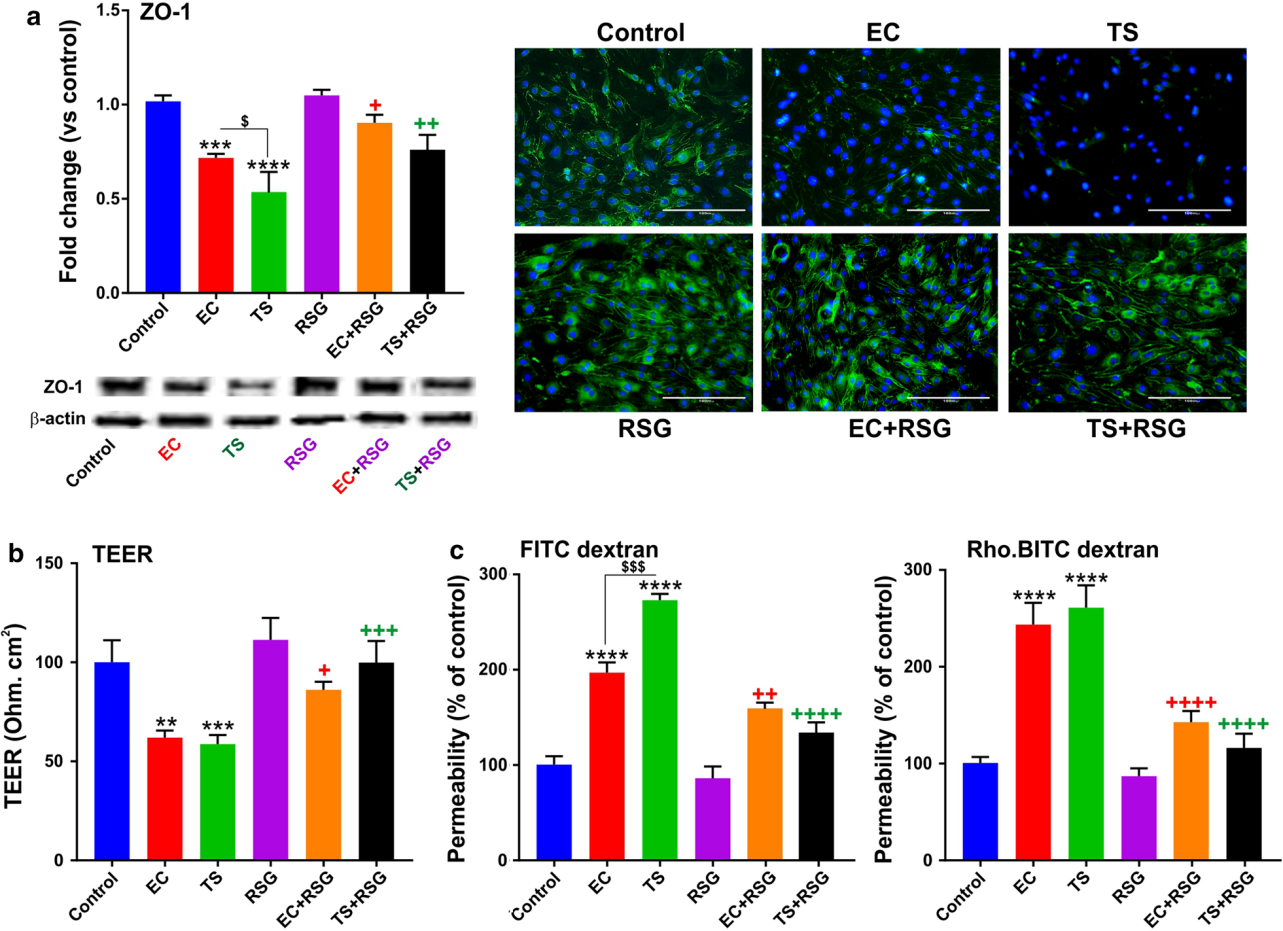
Protective effects of RSG against TS/EC-induced endothelial inflammation and loss of barrier integrity. **a** Immunofluorescence and Western blotting analysis demonstrating downregulation of TJ protein ZO-1 in cells treated with TS/EC and also increased expression of that in the cells including RSG. **b** TEER measurement after treatments demonstrated a significant loss of BBB integrity in cells treated with TS/EC, while RSG prevented the decrease in TEER. **c** Increase in dextran permeability measurements emphasizing the loss of BBB integrity in cells treated with TS/EC was prevented by RSG. n = 3 to 4 biological replicates. **p < 0.01, ***p < 0.001, ****p < 0.0001 versus control; +p < 0.05, ++p < 0.01, +++p < 0.001, ++++p < 0.0001 TS/EC versus TS/EC + RSG; $p < 0.05, $$$p < 0.001 EC versus TS. WB analyses report protein/β-actin ratios

To assess the effect of RSG on BBB integrity, immunofluorescence, Western blot, TEER measurement and labeled dextrans permeability analyses were conducted. As demonstrated in Fig. 4a exposure of mBMEC cells to TS/EC downregulated the expression of the tight junction accessory protein ZO-1 when compared to controls. Notably, these adverse changes were countered by RSG pretreatment. From a functional point of view, TS/EC negatively impacted barrier integrity as demonstrated by TEER measurements (Ω cm^2^) shown in Fig. 4b. The negative impact of both TS and EC on BBB integrity was significantly reduced by RSG. Results were further confirmed by parallel permeability assays using labeled FITC and RITC dextrans demonstrating increased permeability (vs. control) in TS/EC exposed cultures which was reverted following RSG treatment (see Fig. 4c) [30].

## Discussion

Excessive ROS production (endogenous and exogenous) is able to provide a state of redox imbalance causing cellular and tissue damage which are of the main prodromal factors in the onset and progression of several cerebrovascular and neurodegenerative diseases such as stroke, amyotrophic lateral sclerosis (ALS), Parkinson’s disease (PD), Alzheimer’s disease (AD and aging [31–36]. Chronic exposure to TS and EC is a risk factor for vascular endothelial dysfunction. The impact on BBB viability has been recently reported to be primarily linked in a dose-dependent and causative manner primarily linked to ROS, nicotine, and inflammation [8, 9, 16]. The current scientific opinion suggests that ROS/OS play a pivotal role in the pathogenesis of cerebrovascular disorders [17]. Nrf2, as a ubiquitously expressed redox-sensitive transcription factor and the key regulator of redox homeostasis in cells, exerts cytoprotective functions encompassing anti-oxidative and anti-inflammatory responses under physiological conditions [37]. In fact, Nrf2 is a pivotal upstream transcription factor responsible for the regulation of redox balance so that downregulation or suppression of NRF2 activity enhances the cell susceptibility to the detrimental effects of ROS and pro-inflammatory stimuli leading to cell apoptosis [38–40]. Although under normal conditions, Nrf2 is sequestered in the cytoplasm by its inhibitor Keap1, once activated, it translocates to the nucleus and dimerizes with another member of the Cap‘n’Collar family of transcription factors which finally leads to activation of transcription by binding to an antioxidant response element (ARE) located in the promoters of a number of antioxidant genes [24]. Our previous findings have shown that Nrf2 regulates BBB endothelial tight and adherence junction protein expressions whereas chronic exposure to TS and EC adversely impact Nrf2 activity/levels and negatively influence BBB integrity and function [30]. Similarly to chronic smoking/vaping downregulation of Nrf2 worsens the diabetic phenotype and the impairment in endothelial glucose uptake leading to the downregulation of tight junction protein expression and loss of BBB integrity [41, 42]. The results from our previous reports also support an additive release pattern of angiogenic and inflammatory factors by BBB endothelial cells in response to hyperglycemia (HG) with concomitant exposure to cigarette smoke extracts, suggesting the involvement of common pathogenic modulators of BBB impairment [14]. RSG, as a member of the thiazolidinediones (TZDs) family, which is the ligand of the PPARγ, has been currently investigated for the diseases where insulin resistance may be an important factor [24]. For example, Ceolotto et al. tested the effect of RSG on quenching oxidative stress initiated by high glucose by prevention and reduction of NAD(P)H oxidase activation. In fact, They demonstrated that RSG activates AMPK preventing hyperactivity of high glucose-induced NAD(P)H oxidase, possibly by PKC inhibition and thus, protecting endothelial cells against glucose-induced oxidative stress with an AMPK-dependent and a PPARγ-independent mechanism [23]. AMPK has been demonstrated to promote the nuclear accumulation and thus the activity of Nrf2 thus implying that increased expression of PPAR by RSG can be linked to increased activity of Nrf2 and its downstream effectors such as NQO-1 as shown in our work. In another study, Hwang et al. [43] demonstrated that RSG has the potential to reduce vascular oxidative stress rapidly through mechanisms not depending on correction of diabetic major metabolic derangements. Furthermore, Sayan-Ozacmak et al. evaluated the possible impact of RSG on chronic cerebral hypoperfused rats regarding the levels of oxidative stress, reduced glutathione, and hippocampal neuronal damage. Their findings confirmed the beneficial effect of RSG on hypoperfusion-induced hippocampal neuronal damage as a result of the inhibition of oxidative stress [44]. Lee et al. investigated the molecular mechanisms underlying apoptosis initiated by chlorpyrifos (CPF)-mediated oxidative stress and finally they suggest that RSG may employ an anti-apoptotic effect against CPF-induced cytotoxicity by the reduction of oxidative stress and also inhibition of the inflammatory cascade using inactivation of signaling by p38 and JNK, and NF-κB [45]. In a separate study, Kadam et al. observed elevated expression of HO-1 and Nrf2 and downregulated expression of Tlr4 receptor in response to RSG administration when compared to the control group. RSG also prevented preterm birth by downregulating inflammation and upregulating the antioxidant factors NRF2 and HO-1 [46]. Although these studies clearly prove the protective effects of RSG against some other causes of oxidative stress, possible therapeutic use of this drug in the reduction of ROS and BBB integrity prodromal to cerebrovascular disorders could be investigated. Thus, in this preliminary focused study, we assessed the effectiveness of RSG treatment to prevent and reduce BBB damage and OS in response to chronic TS/EC exposure. The results of our study showed that the appropriate RSG dosing based on its impact on cell viability by MTT cytotoxicity (Fig. 1) notably inhibited TS/EC exposure-induced intracellular ROS production confirming cytoprotective effects of RSG (Fig. 2). In order to examine the mechanism underlying the antioxidant effect of RSG, we also detected the effect of RSG on the expression of Nrf2, as one of the major transcription factors regulating the antioxidant defense response and its effector protein downstream signaling molecule NQO-1 which exert acute detoxification and cytoprotective functions [3]. Although TS/EC exposures caused a slight increase in the expression of Nrf2, and also promoted loss of BBB integrity and cellular inflammation (Fig. 3), RSG significantly induced Nrf2 pathway activation and also increased the expression NQO-1 [47]. These results might be due to an antioxidant role of RSG in a PPARγ-dependent manner, leading to the activation of Nrf2 and NQO-1. Our results correlate very well with previous studies by Wang et al. [24] demonstrating that inhibition of PPARγ markedly reduced the protective activity of RSG by preventing Nrf2 activation of the antioxidative response system. Concerning the specific impact on the BBB, our in vitro data (Fig. 4) clearly show that TS/EC exposure does indeed decrease ZO-1 expression in mBMEC cells leading to cerebrovascular impairment in terms of loss of tight junction proteins which was prevented by RSG. As expected, loss of ZO-1 following TS/EC exposure was paralleled by significant TEER decrease and concomitant increase of paracellular permeability to both 4 and 10 kDa labeled dextrans (Fig. 3). Like for ZO-1 expression, Impairments of BBB integrity including loss of TEER and increased permeability to dextrans was reverted by RSG (Fig. 4). Moreover, RSG reduced the expression of the pro-inflammatory adhesion molecule PECAM-1 resulting from TS/EC exposure when compared to untreated TS/EC exposed cultures. This and the protective effect on BBB integrity suggests that RSG could be used to protect the cerebrovascular system from exogenous oxidative stimuli (like those promoted by TS/EC exposure). This preliminary work provides support for further in vivo studies aimed at validating our results and assesses the effectiveness of RSG in more realistic clinical settings (e.g., vascular inflammatory disorders and stroke). Needless to mention that there are limitations inherent to our in vitro study modeling chronic-TS/EC exposure and in vivo studies. The adverse effects of TS/EC observed in the present study may not fully recapitulate those experienced by chronic smokers over a period of months/years which may yield additional side effects that could not be observed in our study. Therefore, clinical/population studies encompassing additional comorbidities (including risk of stroke and other cerebrovascular disorders) will be needed to fully assert the protective effect and feasibility of RSG treatment to reduce and/or prevent the onset of TS/EC-induced cerebrovascular disorders.

In the future in vivo studies, we plan to assess the dose-dependent effect of RSG on C57BL/6J mice by evaluating the vascular endothelial expression levels of the biomarkers we investigated in vitro in the present studies including PPARγ; tight junction expression and topographic distribution of ZO1, occludin and claudin-5, inflammatory biomarkers such as PECAM-1 as well as interleukins. Concerning Nrf2, we will extend the study beyond the expression its level to encompass cellular distribution (cytoplasmic vs. nuclear) in correlation with its downstream targets NQO-1 and the oxidative stress marker HO-1. Additionally, we plan to determine the expression levels of NF-ĸB (nuclear factor kappa-light-chain-enhancer of activated B cells) which is antagonistic to Nrf2 activity [48] and dissect out the mechanistic interrelationship between rosiglitazone, PPARγ pathway and Nrf2 expression/activity. Moreover, the Parameters of oxidative stress such as catalase (CAT), superoxide dismutase (SOD), and plasma malondialdehyde (MDA) will be also assessed to increase the evidence of antioxidant activity of RSG. This is also equally important since antioxidants have been shown in vitro to be relatively effective to counteract TS-induced oxidative stress [49–51]. Additionally, if in vivo study will confirm our results, further experimentation will be carried out to assess whether rosiglitazone could be used to reduce the risk of cerebrovascular injuries not only in current smokers but also in early stage former smokers that remain at a considerable high risk of stroke for several years after quitting.

## Conclusion

In this study, the protective effect of RSG against TS/EC-induced damages was investigated. The pivotal role of Nrf2 in maintaining BBB endothelial structure and functional integrity and healthy cerebrovascular conditions was successfully emphasized. Our data and previous studies suggest that both TS and EC significantly impairs the endothelial function and cerebrovascular condition through the enhanced load of oxidative stress caused by impaired Nrf2 signaling [3–5]. Rosiglitazone notably counteracts these TS/EC-induced adverse effects through modulation of Nrf2 possibly via upregulation of PPARγ expression whereas Nrf2 upregulation was paralleled by the restoration of BBB integrity and reduced endothelial inflammatory responses. In conclusion, RSG could be considered as a promising therapeutic potential to prevent TS/EC induced cerebrovascular dysfunction and possibly other xenobiotic substances which may impact the BBB via oxidative stress-mediated effects.

## Abbreviations

ARE: anti-oxidant response element
BBB: blood–brain barrier
EC: electronic cigarette
CAT: catalase
CSE: cigarette smoke extract
2DM: type 2 diabetes mellitus
FITC: fluorescein isothiocyanate
FTC: federal trade control
HG: hyperglycemia
HO-1: heme oxygenase 1
MDA: malondialdehyde
NF-κB: nuclear factor kappa-light-chain-enhancer of activated B cells
NQO-1: NAD(P)H: Quinone reductase I
Nrf2: nuclear factor erythroid 2-related factor
OS: oxidative stress
PPARγ: peroxisome proliferator-activated receptor gamma
PECAM-1: platelet endothelial cell adhesion molecule-1
RITC: rhodamine B isothiocyanate
ROS: reactive oxygen species
SOD: superoxide dismutase
SCSM: single cigarette smoking machine
TEER: trans-endothelial electrical resistance
TJ: tight junction
TS: tobacco smoke
ZO-1: zonulae occludentes-1
